# Preventing involuntary admissions: special needs for distinct patient groups

**DOI:** 10.1186/s12991-016-0125-z

**Published:** 2017-01-25

**Authors:** Knut Hoffmann, I. S. Haussleiter, F. Illes, J. Jendreyschak, A. Diehl, B. Emons, C. Armgart, A. Schramm, G. Juckel

**Affiliations:** 1 Dept. of Psychiatry, LWL Institute of Mental Health, LWL University Hospital, Ruhr-University Bochum, Alexandrinenstr.1, 44791 Bochum, Germany; 2 Department of Psychiatry, LWL-University Hospital Bochum, Alexandrinenstr. 1, 44791 Bochum, Germany; 3 NRW Center for Health, Gesundheitscampus 9, 44801 Bochum, Germany

**Keywords:** Involuntary admission, Coercion, Legal basics

## Abstract

**Background:**

Coercive measures in psychiatry are a controversial topic and raise ethical, legal and clinical issues. Involuntary admission of patients is a long-lasting problem and indicates a problematic pathway to care situations within the community, largely because personal freedom is fundamentally covered by the UN declaration of human rights and the German constitution.

**Methods:**

In this study, a survey on a large and comprehensive population of psychiatric in-patients in the eastern part of North Rhine-Westphalia, Germany, was carried out for the years 2004–2009, including 230.678 treatment cases. The data were collected from the dataset transferred to health insurance automatically, which, since 2004 is available in an electronic form. In addition, a wide variety of information on treatment, sociodemographic and illness-related factors were collected and analysed. Data were collected retrospectively and analyses were calculated using statistical software (IBM SPSS Statistics 19.0^®^). Quantitative data are presented as mean and standard deviation. Due to the unequal group sizes, group differences were calculated by means of Chi-square tests or independent sample *t* tests. A Bonferroni correction was applied to control for multiple comparisons.

**Results:**

We found an over-representation of involuntary admissions in young men (<21 years) suffering from schizophrenia and in female patients aged over 60 with a diagnosis of dementia. Most of our results are concordant with the previous literature. Also admission in hours out of regular out-patient services elevated the risk.

**Conclusion:**

The main conclusion from these findings is a need for a fortification of ambulatory treatment offers, e.g. sociopsychiatric services or ward round at home for early diagnosis and intervention. Further prospective studyies are needed.

## Background

Since Pinel, involuntary treatment is an important issue for discussion in psychiatry, and today, it is still widely accepted as a necessary part of psychiatric treatment. The highly sensitive and controversial nature of this issue meant that the legal regulations for involuntary treatment in most countries have become very strict. In Germany, there are two main acts that dictate the necessary criteria for involuntarily treatment: the first is the “Psychisch-KrankenGesetz” (PsychKG), and the second relates to guardianship, as defined by the civil law code. The PsychKG mental health act states that patients fulfilling the criteria for involuntary treatment must exhibit a highly acute symptomatology, which is expressed by a high risk of the patient being a danger to themselves or to their environment, including other people. The civil law code relating to guardianship also states that patients should exhibit highly acute symptomatology and thus be at a high risk of being a danger to themselves, but this does not apply to them being a danger to others. Both routes to involuntary admission to mental health services require a medical statement from a psychiatrist as well as an approval from a judge. In this study, we only refer to the PsychKG-based treatments.

Unfortunately, there are appearing reports of increase in the number of involuntary admissions under PsychKG conditions in Germany; similarities are reported from other countries [1–3]. However, there are also reports from Germany which more recently found a large decline between 1988 and 2009 [4], and also [5] could demonstrate, that the overall percentage of involuntary admissions to inpatient wards across the country had stayed relatively stable between 1993 and 2003. Mostly, these findings were interpreted as a result of shortened treatment times and a higher frequency of admissions [6]. A European-wide comparison of compulsory admissions over a period of 8 years stated that Germany had the greatest overall percentage increase (75%), with the UK having the lowest (5%). Germany was also shown to have the second highest rate of involuntary admission per 100,000 inhabitants, with Portugal, France and Denmark having the lowest. According to this study, the most frequent diagnosis was schizophrenia (29.5–52.7%), followed by affective disorders (9.2–13.7%), then substance abuse (5.2–24.5%) and then dementia (2.2–12.6%) [7], which was similar for smaller regions [8–10]; sociodemographic risk factors were suggested to be a married status and living alone. Even in different German hospitals, there was a great variety of coercive measures (1.9–16.2%); also depending on the diagnosis [11], this could also been demonstrated for Switzerland [12]. The incidence of seclusion and restraint varied from 35.6% of all admissions in Austria, to 2.6% in Norway and around 0% in Iceland and the UK, though notably, coercion is legally prohibited in Norway and only 1:1 nursing is used instead [13]. Risk was also elevated on Fridays [14]. In terms of legal status, the change from primarily involuntary to voluntary treatment has been demonstrated to be dependent on a variety of certain sociodemographic factors, such as young age, higher education and being employed [15]. Also rehospitalisation has an effect on the rate of involuntary admission [16]. Severity of psychotic symptomatology, measured by PANSS-scale was a risk factor in two independent studies [17, 18]. In most studies, schizophrenia has the highest impact on involuntary admissions [19, 20], fortified by comorbid substance abuse [21]. Also organic psychosis, married status and young age showed an elevated rate of involuntary admission in New York [22]. Interestingly, in Greece, the F1 diagnosis group showed a reverse risk compared to the risk associated with immigration (53.2 vs. 14.5%), whereas all other diagnostic category groups demonstrated a greater risk [23]. A systematic literature review done in 2008 [24] compared 41 previous studies and concluded that involuntarily admitted patients had a higher suicide rate, but no increased mortality had equal levels of psychopathology and treatment compliance. The primary research goal of this study is to identify factors influencing the risk for involuntary psychiatric hospital admission. Due to the fact that reducing these rates is probably only possible on the background of certain knowledge of these risk factors and changing of supposed structural influence factors depends this knowledge.

## Methods

### Sample

A retrospective, large-scale multicentre comparative study of psychiatric admissions was carried out in the district of Westphalia-Lippe of the German federal state of North Rhine-Westphalia for the years 2004–2009. Data were collected from within the LWL Psychiatry Network that consists of 13 psychiatric hospitals for adults (3700 treatment places). The catchment area covers about 8.5 million inhabitants, thus covering nearly half of the inhabitants of North Rhine-Westphalia and about 10% of the whole German population.

The hospital registry data (§21, German hospital reimbursement law) are usually transferred to health insurance companies as part of the daily routine, thus leading to the accumulation of a reliable and comprehensive database of sociodemographic information on all patients treated in the district. The type of information that is recorded includes date of admission, time of admission, date of discharge, diagnosis, legal status, including changes of legal status during the treatment, all coercive measures used, number of treatments, name of the hospital, date of birth, gender, age, family status, postal code, nationality and religious denomination.

**Fig. 1:**
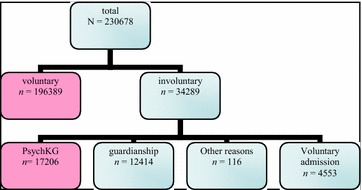
Study-design

The whole sample (*n* = 230.678) was divided into two main subsamples: voluntary (*n* = 196,389, which was 85.14% of the whole sample) and involuntary (*n* = 34.289, 14.86% of the whole sample) admissions to hospital. The involuntary admissions were then further divided into the two subgroups: one that was admitted under the PsychKG act and the other that was admitted on the basis of the civil law code of “guardianship” (Fig. 1). Only the PsychKG cases (17.206 patients; 50.18% of the involuntary admissions, 8.05% of all admissions) were included in the analysis in which PsychKG involuntary admissions were compared to voluntary admissions. The separation of subgroups and the sample size of each subgroup are shown in Fig. 1.

### Data analysis

Data were collected retrospectively and analyses were calculated using statistical software (IBM SPSS Statistics 19.0^®^). Quantitative data are presented as mean and standard deviation. Due to the unequal group sizes, group differences were calculated by means of Chi-square tests or independent sample *t* tests. A Bonferroni correction was applied to control for multiple comparisons.

## Results

Table 1 summarizes the full clinical and demographic data of the voluntary and involuntary subsamples, which are described in the following.

**Table 1:** Sociodemographic and illness-related data of voluntary and involuntary admissions, F0–G3: diagnostic groups according to ICD-10

	Variable	Voluntary (*N* = 196,389), data in %	Involuntary (*N* = 17,206), data in %	Group comparison	*p* value
Gender	Male	57.6	58.0	$\chi_{\left( 1 \right)}^{2} = 1.047$	*p* = .306
	Female	42.4	42.0		
Age	M (SD)	44.41 (17.04)	48.55 (19.52)	t_(19,561.444)_ = −26.877	*p* < .001
	<18	0.1	0.1	$\chi_{\left( 7 \right)}^{2} = 1263.41$	*p* < .001
	18–21	5.4	6.2		
	22–30	18.0	13.6		
	31–40	22.1	18.5		
	41–50	24.5	22.7		
	51–60	12.7	12.3		
	61–70	6.9	8.4		
	>70	10.2	18.1		
Marital status	Married	28.4	30.7	$\chi_{\left( 2 \right)}^{2} = 195.049$	*p* < .001
	Single	71.6	69.3		
Nationality	German	95.1	94.0	$\chi_{\left( 4 \right)}^{2} = 39.927$	*p* < .001
	Turkish	2.2	2.4		
	Polish	0.3	0.4		
	Italian	0.3	0.3		
	Other	2.1	2.8		
Religion	Roman-Catholic	49.6	54.2	Due to a high amount of missing data no statistics could be used	
	Protestant	34.4	32.7		
	Muslim	3.9	3.1		
	Other	12.1	10.0		
Month of admission (most frequent)	1	August: 8.7	July: 9.5	$\chi_{{\left( {11} \right)}}^{2} = 88.221$	*p* < .001
	2	July: 8.6	August: 9.1		
	3	March: 8.6	June: 9.0		
Time of day of admission	Morning (8–12 a.m.)	48.0	16.2	$\chi_{\left( 2 \right)}^{2} = 9704.74$	*p* < .001
	Afternoon (12 a.m.–4 p.m.)	27.2	26.3		
	Late afternoon and night (4 p.m.–8 a.m.)	24.8	57.5		
Admission type	Hospital doctor on call	13.7	33.5	$\chi_{\left( 6 \right)}^{2} = 3449.047$	*p* < .001
	(Psychiatrist)	31.5	31.8		
	General practitioner	46.1	19.7		
	Other specialist	8.4	14.7		
Service allocation	General psychiatry	40.4	48.2	$\chi_{\left( 3 \right)}^{2} = 2380.468$	*p* < .001
	Addiction psychiatry	41.3	28.0		
	Gerontopsychiatry	11.8	20.9		
	Other	6.5	3.0		
Main diagnosis (ICD-10)	F0	5.1	16.1	$\chi_{{\left( {10} \right)}}^{2} = 7709.130$	*p* < .001
	F1	36.7	22.8		
	F2	14.7	30.5		
	F3	28.2	14.9		
	F4	3.1	2.8		
	F5	0.2	0.1		
	F6	5.2	6.8		
	F7	1.7	1.8		
	F8	0.0	0.0		
	F9	0.2	0.5		
	G3	2.2	3.4		
Comorbidity	Psychiatric	47.4	43.6	$\chi_{{\left( {22} \right)}}^{2} = 1137.372$	*p* < .001
	Somatic	10.8	10.7	$\chi_{{\left( {40} \right)}}^{2} = 208.934$	*p* < .001
Duration of stay	M (SD)	22.60 (23.795)	24.84 (28.480)	t_(19,367.606)_ = −10.005	*p* < .001
	1 day	1.4	3.7	$\chi_{\left( 8 \right)}^{2} = 5922.69$	*p* < .001
	2 days	5.0	10.0		
	2 weeks	42.1	34.0		
	6 weeks	36.4	34.2		
	≥7 weeks	15.1	18.2		
Number of previous treatments	M (SD)	7.61 (12.79)	5.84 (10.12)	t_(22,316.321)_ = 21.397	*p* < .001
	1	24.5	33.5	$\chi_{\left( 4 \right)}^{2} = 786.410$	*p* < .001
	2	16.2	16.3		
	3	11.0	10.4		
	4–10	29.6	25.7		
	>10	18.8	14.1		

### Demographic factors

Mean age was 4.12 years younger in the involuntary group (*p* < .001). Clustered age groups compared due to the legal implications showed a highly significant over-representation of the age groups 18–21, 61–70 and over 70 for involuntary admission. A comparison regarding the change of average age at time of both voluntary and involuntary admission over the time of study (Table 2) showed a steady rise from 43.73 years in 2004 (SD = 17.03) to 45.33 years (SD = 17.48) in 2009, demonstrating a 3.7% increase, but no significance (*p* = .306).

**Table 2:** The mean (M) age of admissions in the voluntary and involuntary group for each year over the study period of 2004–2009

Year of admittance	Age voluntary		Age involuntary	
	M	SD	M	SD
2004	46.66	18.569	43.73	17.025
2005	47.71	19.314	44.01	17.077
2006	48.06	19.571	44.22	16.936
2007	48.89	19.744	44.28	16.799
2008	50.16	19.805	44.65	16.862
2009	49.46	19.794	45.33	17.476

Married people appeared to display an increased risk for involuntary admission (married 28.4 vs. 30.7%, single 71.6 vs. 69.3%, *p* < .001).

Nationality showed no significant risk related to involuntary admissions. However, it is important to note that the data did not provide sufficient information to identify migration status, especially for those who were second or third generation migrants. Analysis of age-related subsamples showed that 38.5% of the patients with German origin were between 22 and 40 years old, and the fraction of patients from a non-German origin in this age group was higher than in other age groups (53.3% Italian, 54.3% Polish, 69.4% Turkish). From looking at the legal status of patients in the age group of 22–40, it appears that the portion of the voluntarily treated Germans and Italians was higher than those that were involuntarily treated (39.1 vs. 30.5% for the German population, 54.5 vs. 43.2% in the Italian group), 69.5% of the group with Turkish origin voluntarily treated were between 22 and 40 years old, whereas in the group of the involuntary treated patients, this amount was a little bit higher. A very clear difference between voluntary and involuntary admissions could be seen for the patients with Polish origin, whereby the proportion of those in this age group who were voluntarily treated was 53.3 and 69.6% in the involuntarily treated group.

Due to a large quantity of missing data, the influence of religious denomination is not interpretable.

### Service-related factors

The total number of admissions for each month was calculated and a group comparison was made for the three months that had the highest number of admissions for each group. In the voluntary group, August (8.7%), July (8.6%) and March (8.6%) showed the highest number of admissions. The highest number of admissions in the involuntarily treated patients was in July (9.5%), followed by August (9.1%) and then June (9%). This difference showed high statistical significance (*p* < .001). One remarkable finding was that the most involuntary admissions happened during the summer months (24.7 vs. 27.6%, *p* < .001).

Further on, the day was divided into three parts: morning, afternoon and late afternoon and night. The greatest number of voluntary admissions (48%) occurred in the morning (8–12 a.m.), whereas only 16.2% of involuntary admissions occurred at this time of the day. The time of day in which the greatest number of involuntary admissions occurred was in the late afternoon and night, i.e. between 4 p.m. and 8 a.m. (57.5%), though only 24.8% of the voluntary admissions happened at this time (*p* < .001).

The groups differed also highly significant regarding the admitting profession: voluntary patients were primarily admitted by non-psychiatrists (54.5%, general practitioners: 46.1%, other specialities: 8.4%). In the involuntary group, general practitioners admitted 19.7% of the patients, other specialities for 14.7% (total 34.4%). Psychiatrists in practice have quite equal representation in both groups (31.5 vs. 31.8%). The involuntary group showed a higher amount of admissions initiated by hospital psychiatrist on call (33.5 vs. 13.7%, *p* < .001). Also the receiving department was of great importance for the investigated question. The largest proportion of the voluntarily treated patients was admitted to a department for addiction (41.3%), whereas most of the involuntary admissions were allocated to general psychiatry (48.2%). The voluntary admissions were allocated to gerontopsychiatry (11.8%) less than the involuntary admissions (20.9%, *p* < .001).

### Illness-related factors

The most common diagnosis in the voluntary group was substances abuse (36.7%), while in the involuntary group, the most common diagnosis was in the schizophrenia (30.5%); schizophrenia was less than half as common in the voluntary group (14.7%). In the group of affective disorders (F3), there were quite reverse findings (28.2 vs. 14.9%), further differentiation (i.e. to F30, F31, F32, F33) showed an excess of bipolar affective disorder (18.9 vs. 7.2%) in the involuntary group.

In the ICD-10 F0/G3 diagnostic category (organic psychiatric disorders, in particular, dementia and other neurodegenerative diseases), a substantial difference was revealed between the groups. 19.5% of the involuntary group had a F0/G3 diagnosis, but only 7.3% of the voluntarily treated patients had this diagnosis.

For personality disorders (F6), a mild over-representation of involuntarily treated patients was found (6.8% involuntary vs. 5.2% voluntary). Group differences were highly significant for the main categories of diagnosis (*p* < .001).

Furthermore, the changes in percentages of diagnoses over the study period were different between the diagnostic categories (see Table 3). While most ICD-10 categories stayed quite stable (F0, F2, F5, F6, F7), a decline of ICD-10: F4-cluster could be demonstrated, whereas dementia (ICD-10: F0, G3) and affective disorder (ICD-10: F3) showed a clear increase (F0: 14.7% to 21.7%, F3 13.2% to 16.8%).

**Table 3:** Percentage and number of admissions in each ICD-10 diagnostic category over study period, both groups

Main diagnosis	Year											
	2004		2005		2006		2007		2008		2009	
	*N*	%	*N*	%	*N*	%	*N*	%	*N*	%	*N*	%
F0	324	12.8	406	13.9	446	15.7	471	16.6	567	18.8	563	18.5
F1	683	26.9	693	23.7	646	22.7	604	21.3	630	20.9	659	21.6
F2	818	32.2	957	32.7	834	29.4	868	30.6	877	29.0	900	29.5
F3	336	13.2	408	13.9	453	16.0	426	15.0	436	14.4	512	16.8
F4	102	4.0	84	2.9	76	2.7	79	2.8	78	2.6	58	1.9
F5	1	0.0	3	0.1	4	0.1	1	0.0	3	0.1	2	0.1
F6	170	6.7	196	6.7	188	6.6	195	6.9	222	7.4	204	6.7
F7	34	1.3	52	1.8	53	1.9	49	1.7	59	2.0	56	1.8
F8	0	0.0	2	0.1	0	0.0	0	0.0	0	0.0	0	0.0
F9	13	0.5	10	0.3	23	0.8	21	0.7	17	0.6	9	0.3
G3	48	1.9	106	3.6	109	3.8	111	3.9	124	4.1	81	2.7
Not stated	9	0.4	10	0.3	8	0.3	7	0.2	7	0.2	5	0.2
Total	2538	100	2927	100	2840	100	2832	100	3020	100	3049	100

Psychiatric comorbidity was higher in the voluntarily treated group (47.4 vs. 43.6%, *p* < .001), whereas somatic comorbidity was quite equal (10.8 vs. 10.7%).

The duration of stay was compared in two different ways. First, a comparison of the median duration of stay was carried out, which revealed that the voluntarily treated patients had a generally shorter median duration of stay of 22.6 days (SD = 23.80) relative to the median duration of stay of the involuntary group (24.84 days, SD = 28.48, *p* < .001). In a second step, a comparison of both groups regarding different clusters of durations of stay was performed, looking at the proportion of patients that stayed for 1 day, 2 days, 2 weeks, 6 weeks and 7 or more weeks. The first notable result was that more than twice as many of the involuntarily admitted patients, as compared to the voluntary group, were discharged on the day of admission (3.7 vs. 1.4%). These findings were also very similar for duration of stay of 2 days (10 vs. 5%). However, medium-length stays were more common for the voluntary rather than involuntary admissions, as the proportion of the voluntary group that stayed for 2 and 6 weeks was 42.1 and 36.4%, respectively, whereas the proportion of stays for 2 and 6 weeks was 34.0 and 34.2% for involuntary admissions. For longer term stays (>7 weeks), the involuntary group was 18.2, 15.1% in the voluntary group. To sum up, it could be demonstrated that very short treatment times (1–2 days) were more common for involuntary admissions (6.4 vs. 13.7%, *p* < .001).

The mean number of previous treatments received by patients differed significantly in both groups [voluntary 7.61 (SD = 12.79), involuntary 5.84 (SD = 10.12), *p* < .001]. Looking at clustered numbers of pre-treatments, a further comparison of the groups was made. A substantially greater number of involuntary admissions were first hospitalized (24.5 vs. 33.5%), whereas the difference between the proportion of voluntary and involuntary groups for their second and third treatments was minimal (16.2 vs. 16.3% for 2nd treatment, and 11 vs. 10.4% for 3rd treatment). For the patients who had received 4–10 previous treatments, a greater proportion was represented by voluntary admissions (29.6 vs. 25.7%), which was also the case for those that had received more than ten previous treatments (18.8 vs. 14.1%).

## Discussion

Analysis of the relationship between legal status, gender, age and diagnosis showed that the largest proportion of involuntary admissions was with men between 22 and 30 years old who suffered from an ICD-10: F2 diagnosis, which is concordant with the literature findings. It is likely that schizophrenia is a diagnosis that still has insufficient services for early recognition, and therefore schizophrenia appears in many cases with very impressive psychopathological features and thus may lead to a higher rate of emergency admittances. On the other hand, there is a large representation of women aged over 70 with an ICD-10: F0/G3 diagnosis. The predominance of females in this older group is likely to be an effect of the longer life expectancy of woman (actual data in Germany: woman: 82.73 years, man 77.72 years [25]. What also could be displayed was a clear decline in the ICD-10: F4-cluster which may be explained by an improvement of the out-patient service structure for people getting into acute psychiatric crisis situations.

The finding that being married is possibly associated with an increased risk for involuntary admission is also quite interesting and was not expected, particularly as stable social surroundings are generally thought to be protective. Possibly, additional problems that accompany being in a relationship (e.g. marital crisis, problems in education of children, deaths of close relatives) may act as stress factors which may worsen illness. Previous findings of the influence of being married on preventing involuntary admission are mixed in literature, and still not satisfactorily elucidated [22, 26, 27]. Further research on this topic is needed, especially as only a few studies on this topic exist until now [10, 20, 22]. Therefore, it is likely that the influence of family status has a multidimensional aspect and thus could only be clarified in a specific, prospective survey with a main focus on this topic.

Another quite interesting finding is that most of the voluntarily treated patients were admitted during regular hospital hours rather than on duty time. One explanation for this finding could be that the more severely ill patients are less able to admit themselves to hospital, and only the presentation of acute symptomatology like suicidal intent or aggressive behaviour would initiate an admission. This might indicate that the needs of severely affected psychiatric patients are not met by the current structures of psychiatric services. This was confirmed by other studies [28].

Regarding the migration background, the most interesting finding shows that the rate of patients with a non-German origin was higher in the involuntary than in the voluntary group, which is especially prominent for migrants coming from eastern European countries [9]. The high representation of young Polish people for involuntary admissions may be explained by the long history of immigration to this area for work in the coal mines over the past 100 or so years, which now has gone down. In general, the findings from our study show that young people who had immigrated to North Rhine-Westphalia seemed to be especially more often in psychiatric crisis. Problems of integration probably have an important influence on this effect, possibly also due to communication barriers [29, 30].

Due to the result that most of the involuntarily admitted patients were diagnosed with schizophrenia spectrum disorders (F2), and were also mostly admitted during the summer months, the question of whether there may be a relationship between environment, climate and psychosis should be raised again. Many authors have suggested that there is the potential for higher dopamine levels during the summer months due to higher environment temperatures [31–34]. Another possible explanation for this season-related finding could be that psychiatric services during summer months are not quite as well equipped as in other seasons, due to a higher number of staff taking holidays, and thus leading to a reduction in resources. It may be that access to the patients’ support system, like guardianship, during the summer months may be reduced, as compared to the other seasons. However, this suggestion is highly speculative and would require confirmation from future studies looking more specifically into the effect of season on involuntary admissions.

Another main finding from this study was that a higher proportion of involuntary admissions fell under the category of short-term treatments, when compared to voluntary admissions. One possible explanation for the higher incidence of short-term treatments in the involuntary group may be due to the over-representation of the diagnosis of acute intoxication (ICD-10: F1X.0) in this group, which normally leads to only very short treatment times due to the short duration of the influence of intoxication on psychopathology.

## Conclusion

Due to limitation of a retrospective study with a restricted and unswayable data pool, our findings have to be considered carefully. Our main findings, the risk for involuntary admissions for older women with dementia and younger man with schizophrenia lead to the necessity of special attention for these groups of people. Further on, a prospective study is needed to minimize the limitations reported below.

## Limitations

To the best of the authors’ knowledge, this study is the most extensive investigation on this topic related to a catchment area of this size, but was limited by the structure of the data, because the data structure had been already predefined for purposes other than research, and could not be adjusted for the use in such a study. This has to be taken into account when considering its results: the use of complete hospital admission and discharge registers enabled us to investigate the largest cohort of this kind in psychiatric hospitals so far published. However, at the same time, the retrospective nature of this study and the structure of the data only allowed for limited conclusions, partly as a result of the limited period in which the data covered, i.e. covering data on admissions between 2004 and 2009. This was due to the fact that these data had only been stored electronically since 2004. In addition to this, information on the potential risk of harm to self or others prior to the admission or the administration of medication throughout the hospital stay was not available, otherwise additional connections and conclusions could have been drawn out of such data. Other factors (e.g. family background, physical and cognitive development, suicidality, aggressiveness, treatment variables or past experiences regarding the health care system) that may have had an influence on the admission status were also not included. In order to draw a realistic picture of the current mental health care situation, and to consider the severity and course of certain diseases, all treatment data over the 6-year period were used, and as a result, it was not that data on single patients were included in the analysis, but rather treatment “cases”. One patient might therefore have been included several times if they had been re-admitted during the study period. This might have thus caused a bias in the results on age, gender and diagnosis in the subgroups. The very important issue of patients’ subjective perception towards coercion during admission could also not be assessed because of the retrospective nature of the data used and the large sample size. Further research on this topic is evidently needed, ideally in the form of a prospective study.

Despite the limitations, the following issues were identified as areas that could be addressed to reduce involuntary psychiatric treatments. The first issue highlighted by this study that could have an influence is the early recognition and prevention of psychotic disorders in young people, with a particular focus on improving these services for males. In addition, a greater involvement of government mental health services might be very useful in preventing acute psychiatric crises and thus also reducing consecutive involuntary admission. Secondly, this study highlights the importance of making service improvements for older people, especially females, suffering from delirium/dementia. Due to the overall increasing age of the population, it is inevitable that the number of older people with ICD-10 F0/G3 diagnosis will rise in both genders. Only an improvement of non-hospital psychiatric services in conjunction with early intervention services could help to prevent this group of patients from involuntary treatment. Thirdly, in terms of immigration, the data in this study particularly identify an over-representation of Polish people being involuntarily admitted. One step towards addressing this issue of immigration as being a possible risk factor for involuntary psychiatric treatment may be to have a closer look at improving the integration of immigrants, while also taking addiction and addiction prevention into special consideration.
